# The implementation of pharmaceutical services in public hospitals in Mexico: an analysis of the legal framework and organizational practice

**DOI:** 10.1186/s40545-021-00318-7

**Published:** 2021-05-05

**Authors:** Laura C. Vargas López, Fela Viso Gurovich, Anahí Dreser Mansilla, Veronika J. Wirtz, Michael R. Reich

**Affiliations:** 1 Faculty of Pharmacy, Instituto de Ciencias de la Salud, Autonomy University of Hidalgo State, Carretera Pachuca-Actopan camino a Tilcuautla s/n Pueblo San Juan Tilcuautla, 42160 Hgo, Mexico; 2 Pachuca, Mexico; 3 Center of Health Systems Research, National Institute of Public Health, Avenida Universidad 655, Santa María Ahuacatitlán, 62100 Cuernavaca, Morelos México; 4 Department of Global Health, Boston University School of Public Health, 801 Massachusetts Avenue, Boston, MA 02118 USA; 5 Department of Global Health & Population, Harvard T. H. Chan School of Public Health, 677 Huntington Ave, Boston, MA 02115 USA

**Keywords:** Health services, Hospitals public, Institutional and organizational analysis, Legislation pharmacy, Mexico, Pharmaceutical services, Pharmacy service hospital

## Abstract

**Background:**

The implementation of pharmaceutical services in hospitals contributes to the appropriate use of medicines and patient safety. However, the relationship of implementation with the legal framework and organizational practice has not been studied in depth. The objective of this research is to determine the role of these two factors (the legal framework and organizational practice) in the implementation of pharmaceutical services in public hospitals of the Ministry of Health of Mexico.

**Methods:**

Semi-structured interviews were conducted with four groups of actors involved. The analysis focused on the legal framework, defined as the rules, laws and regulations, and on organizational practice, defined as the implementation of the legal framework by related individuals, that is, how they put it into practice.

**Results:**

The main problems identified were the lack of alignment between the rules and the incentives for compliance. Decision-makers identified the lack of managerial capacity in hospitals as the main implementation barrier, while hospital pharmacists pointed to poor regulation and the lack of clarity of the legal framework as the problems to consider.

**Conclusions:**

Although the legal framework related to hospital pharmaceutical services in Mexico is inadequate, organizational factors (such as adequate skills of professional pharmacists and the support of the hospital director) have facilitated gradual implementation. To improve implementation, priority should be given to evaluation and modification of the current legislation along with the development of an official minimum standard for activities and services in hospital pharmacies.

**Supplementary Information:**

The online version contains supplementary material available at 10.1186/s40545-021-00318-7.

## Background

The analysis of health services implementation is a complex process that requires a multifactorial approach [1–3] and taking a medium- to long-term perspective [4–6]. Analyzing the heterogeneity of implementation processes should be done from various perspectives, and should include the context of implementation as a key element of the process [2, 5, 7]. Contextual elements include the needs and interests of the actors [7, 8], the design of the health system [9] and the implementation of the legal framework for the fulfillment of specific objectives [10–13]. The present study analyzes contextual factors that affect the implementation of pharmaceutical services in public hospitals in Mexico.

Mexico´s health system is divided into a public health system and a private one. The public national health system is divided into social security institutions, which provide health services to salaried workers, and public services provided by the Ministry of Health (MoH) for workers of the informal sector, self-employed and their families. MoH facilities include second- and third-level hospitals run by subnational authorities, as well as highly specialized hospitals run by MoH central authorities. Until the end of 2018, users of MoH services could opt for a public insurance program called Seguro Popular that covered around 53 million registered people and their families (42% of the population) [14, 15]. This program allowed people and their families to have access to specified health services: free essential medicines, vaccines, medical services, hospital services, some surgery services and pharmaceutical services. Seguro Popular included a separately financed system of specified medicines for expensive treatments (e.g., children´s cancer, HIV) known as Fondo de Protección contra Gastos Catastróficos. During the period 2012–2018 all states in Mexico had an agency responsible for the stewardship of Seguro Popular as part of a decentralized model of national healthcare [16]. In 2019, Seguro Popular was eliminated and a replaced by a new institution called INSABI (Instituto de Salud para el Bienestar, or Institute of Health for Welfare).

In Mexico, social security institutions function in a conservative framework, which delays the introduction of new ideas [17]. On the other hand, MoH hospitals have been more receptive to changes led by public policies, including the new concept of Hospital Pharmacy and the implementation of Hospital Pharmaceutical Services in their second- and third-level hospitals. This greater acceptance is the main reason why this research is focused on hospitals of the MoH.

Hospital Pharmaceutical Services (HPS) represent an organizational opportunity for hospitals to raise the quality of care through the appropriate use of medicines and patient safety [18–24]. Therefore, several countries have focused on HPS implementation [25] with the support of international organizations [26–32].

In Mexico, the implementation of HPS in MoH public hospitals began in the 1990s [33–35]. In the following years, several organizations promoted university education and training of human resources focused on HPS, especially patient-oriented pharmacy services [36]. Some pharmacy associations provided continuous professional education [37–40]. In 2010, the National Model of Hospital Pharmacy, a service delivery model proposed by the Mexican MoH, was developed.

Regarding the legal framework that regulates the practice of HPS in Mexico, we present the key milestones from 1983 to 2018 in the chronology shown in Table 1 and a more detailed description in Additional file 1: Annex 1.

**Table 1:** Chronology of key health policy and legal framework events related to hospital pharmacy in Mexico

Year	Legal framework events
1983	Political Constitution of the United Mexican States^a^
1984	The General Health Law and its modifications^b^ (Health Protection is established in Article 4 of the Political Constitution of the United Mexican States, from which the General Health Law derives, as well as the regulations and the Mexican official norms. All of them determine the legal compulsory framework to which are subordinated all the services, institutions and products that have a relationship with the population´s health.)
1988	Pharmacopeia of the United Mexican States 5th edition, updated and published every 5 or 6 years; in 2018 the 12th edition was published^b^ (Determines the quality standards for the production and storage of health goods)
1992	First publication of the Federal Law of Metrology and Normalization^b^
1995	Opening of the Centro Nacional de Farmacovigilancia y Tecnovigilancia. (This Center complies with the requirements of WHO International Collaborative Center reporting Adverse Drug Reactions in the Mexican population.)^c^
1997	First FEUM Supplement for pharmacies, drugstores, apothecaries and warehouses for the storage and distribution of medicines^d^
1998	Publication of the regulation of health products (RIS)^d^
2002	First publication of the Official Mexican Norm NOM-220-SSA1-2002 Installation and operation of pharmacovigilance^e^
2004	COFEPRIS established as the regulatory body for pharmacies^d^ (It is a federal agency of the government of Mexico, which according to the General Health Law carries out regulation, control and health promotion of, among other things, health establishments and medicines)^f^
2005	Publication of the FEUM Supplement for establishments dedicated to the sale and supply of medicines and other health goods, third edition^d^
2007	Health Sector Plan 2007–2012^d^
2010	National Model of Hospital Pharmacy^d^
	Publication of the FEUM Supplement for establishments dedicated to the sale and supply of medicines and other health supplies, fourth edition^d^
2012	Updating of Official Mexican Norm NOM-220-SSA1-2012 Installation and operation of pharmacovigilance^e^
	The General Health Council published the standards for the Certification of Hospitals, following the International Joint Commission for Accreditation of Hospitals recommendations, adapting them to the needs and the characteristics of Mexican hospitals
2014	Publication of the FEUM Supplement for establishments dedicated to the sale and supply of medicines and other health goods, fifth edition^d^
2015	Publication of the Standards for the certification of hospitals by the General Health Council^d^
2016	Updating of Official Mexican Norm NOM-220-SSA1-2016 Installation and operation of pharmacovigilance^e^
2018	SiNaCEAM patient safety model. Standards to implement the model in hospitals^d^
	Publication of the FEUM Supplement for establishments dedicated to the sale and supply of medicines and other health goods, sixth edition^d^

COFEPRIS: Federal Commission for the Protection against Health Risks for its Spanish acronym. FEUM: Pharmacopeia of the Mexican United States for its Spanish acronym. RIS: Regulation of health products for its Spanish acronym. SiNaCEAM: National Certification System for Healthcare Establishments

^a^Primary law, ^b^Secondary law, ^c^Regulatory Institution, ^d^Manuals or procedures derived from secondary laws, ^e^Official Norm

^f^COFEPRIS was an autonomous entity of the Ministry of Health with technical, administrative and operation autonomy. Its main functions: assess health risks and exercise health control and surveillance for the use and consumption of goods and services, health supplies, health emergencies and the provision of health services

The main objective of this study was to gain a better understanding of the implementation of Hospital Pharmaceutical Services in Mexico in MoH public hospitals by focusing our analysis on two factors: the legal framework, which is defined as the related rules, laws and regulations, and organizational practice, which is defined as the implementation of the legal framework by organization and related individuals, that is, how they put it into practice. Additionally, we use our analysis to identify important obstacles and opportunities in order to make recommendations for improvement.

## Methods

### Conceptual framework

**Fig. 1:**
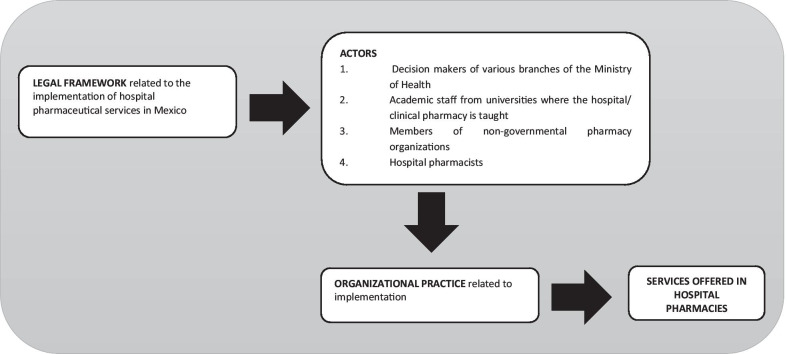
Conceptual framework for this analysis of HPS implementation

We developed the conceptual framework for this analysis of HPS implementation, as presented in Fig. 1. In our analysis, we take the legal framework as the starting point. We then analyze how different actors organize themselves for HPS implementation in the real world, that is, how the legal framework is put into organizational practice. Each of its components is described below.

### Hospital pharmaceutical services

Based on the definitions proposed by Moullin et al. [41] and the Pan American Health Organization [31], we define hospital pharmaceutical services as the set of actions provided by a pharmacist to the patient, the outpatient population or another health professional. These actions are organized and carried out by the pharmacy service of a hospital, through the application of pharmaceutical and health knowledge, with the ultimate goal of raising the quality of care. HPS are implemented according to the needs and characteristics of each hospital.

### The role of legal framework and organizational practice in implementation

When thinking of the health system as an organized social response to the health needs of a population, the importance of the actors involved is evident. Understanding their position, power, needs, and interests are key tools in implementation analysis [7]. One way to encourage these actors to implement evidence-based practices is by generating the correct incentives, for example, economic benefits, to encourage the application of rules and compliance with legal regulations [42, 43].

Likewise, health systems can be seen, in part, as institutions guided by norms, laws and regulations, whose purpose is to coordinate the actors involved in the production of health results [4, 43]. In this document, we use the term “legal framework” to refer to the rules, laws and regulations related to HPS. In many countries, the legal framework of health systems is not adequate due to the lack of sufficient incentives that favor the appropriate performance of health personnel [44, 45].

The role of organizational practice in implementation can be seen in the way in which organizations choose to implement and comply with standards is conditioned by their organizational capacity [46]. According to Mathauer and Carrin, low organizational capacity may be due to: "lack of leadership, human resources without adequate skills, lack of financial resources, poor infrastructure or inappropriate organizational procedures and structures" [46].

### Gathering information

This study focused on the hospitals of the Mexican MoH. Semi-structured interviews were conducted with key informants from August 2018 to March 2019. The first author (LV: pharmacist, PhD student) conducted all the interviews in Spanish and stated to the participants her position as one of the stakeholder groups: pharmacists. LV received qualitative research training during doctoral studies. During the interviews, only the participant and the researcher were present. One interview per participant was conducted. At the beginning of the interview, oral informed consent was carried out and the informants' anonymity was guaranteed. The interviews were audio recorded and transcribed and all interviews were transcribed complete. Analysis findings were discussed with co-authors.

We carried out two types of sampling. Participants were selected by intentional sampling to include all representatives of the following groups: (1) decision-makers from federal government agencies related to the governance and regulation of pharmacies in hospitals of the MoH; (2) staff from non-governmental organizations directly related to hospital pharmacy in the country; and (3) academic staff from the main universities where training in clinical pharmacy is taught. In addition, a group of hospital pharmacists was formed who were selected by snowball sampling due to the larger size of this population.

Semi-structured interviews were conducted around four themes: (1) regulation of pharmacies in hospitals; (2) perceptions of the implementation of pharmaceutical services; (3) the main actors involved; and (4) the opportunities and barriers for the implementation of hospital pharmaceutical services (Table 2). No specific questions were asked, but the informant was allowed to develop the topics freely, as long as their perspective on the four topics mentioned was included in interview guide (Additional file 2: Annex 2). The final sample size was decided based on the objective to interview at least one decision-maker from each federal institution related to the implementation of HPS and a representative from each of the three main related NGOs. For hospital academics and pharmacists, the sample size was established by theoretical saturation (the information provided on each of the interview topics began to be repeated, no new relevant information emerged).

**Table 2:** Characteristics of the 25 informants

Gender	Male	12
	Female	13
Decision-makers	Representatives of the MoH (COFEPRIS, General National Council, General Direction of Quality and Health Education of the Federal MoH, General Direction of planning and development in health of the Federal MoH)	5
	Experts of the Permanent Commission of the FEUM	2
Non-governmental organization staff	Members of colleges and associations of hospital pharmacists	3
Academic staff	State universities	3
	National universities	1
	Private universities	1
Hospital pharmacists	Tertiary level hospitals of the MoH	5
	Secondary level hospitals of the MoH	3
	Private hospitals (tertiary level hospitals)	2

### Data analysis

**Fig. 2:**
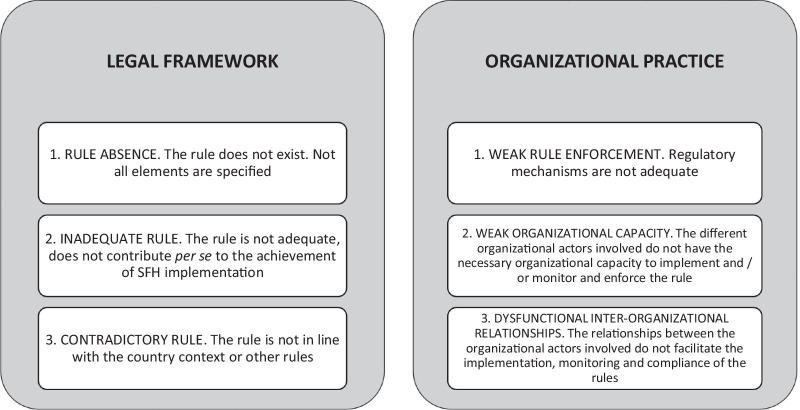
Categories proposed by Mathauer and Carrin for the analysis of the legal framework and organizational practice

The interviews were analyzed by the main author (LV), using the coding of the transcription line by line, until the information in all the categories of analysis proposed by Mathauer and Carrin were extracted from the information contained in each interview (Fig. 2).

As mentioned previously, Mathauer and Carrin propose six categories of analysis, which they call bottlenecks, to address the relationship between the legal framework and organizational practice (Fig. 2) [46]. Their analytical perspective has been used in other countries to evaluate the health financing system [12], as a tool for planning and developing policies [11], and for investigating barriers and opportunities in policy creation [13]. In this article, we use these six categories for the analysis of the implementation of health services, specifically the HPS.

A total of 25 interviews were conducted with actors from nine different states in Mexico (Hidalgo, Mexico City, Baja California, Nuevo León, Guanajuato, State of Mexico, Campeche, Puebla, and Jalisco) were conducted, including seven federal decision-makers from the MoH, four academics from various different public universities and one from a private university, ten hospital pharmacists, and three members of the principal non-governmental organizations related to hospital pharmacy. Because other informants pointed out the importance of interviewing pharmacists from private hospitals, the two most frequently mentioned pharmacists from private hospitals were included. Table 2 summarizes the characteristics of the key informants.

The interviews lasted an average of 43 min (median = 34 min) and were mainly conducted face-to-face [12], although some were also conducted by telephone (seven) and by Skype (four). These interviews were carried out electronically because the informant requested it or by mutual agreement considering convenience due to long travel distance.

Analysis of the data was carried out following a constructivist perspective, using the framework method (FWM) [47]. The FWM is a qualitative content analysis method, developed for use in large-scale policy research. FWM uses a spreadsheet approach. Its defining characteristic is the matrix output: rows (cases), columns (codes), and cells of summarized data. Its structure allows the data to be systematically reduced to analyze them by case and by code, since the opinions of each research participant are connected to other aspects of their story within the matrix [47]. For the writing of the results report, the consolidated criteria for reporting qualitative research (COREQ) were considered. Additional file 3: Annex 3. [48].

### Ethical aspects

The project was reviewed and approved by the Research Ethics Committee of the National Institute of Public Health of Mexico with registration before Conbioética: 17CE100420160708 and Cofepris: 13 CEI 17 00736 AND FWA: 0015605. To carry out the oral informed consent process, the main researcher informed all participants, by telephone, about the objective, the scope of the investigation, as well as the anonymous and voluntary condition of their participation. In addition, contact details were provided to ensure further communication if necessary.

## Results

HPS implementation faces a variety of institutional and organizational bottlenecks that affect future progress. Although there is some legislation that governs HPS implementation in Mexico, a series of conflicting rules or unresolved problems exist. The main problems identified in this study were the lack of alignment between different rules and the few incentives for organizations and individuals to comply with the existing rules.

Differences were found in the perception of compliance with the regulations and their implementation among the different actors. On the other hand, agreements were also found between the actors; for example, most of the actors interviewed in this study agreed that the support of the hospital director and the head of the area (administrative or clinical) favors the successful implementation of HPS. Table 3 describes the results in summary and then describes the results obtained in each of the analysis categories.

**Table 3:** Stakeholders' perceptions of the legal framework and organizational practice, related to implementation problems

Category	Actors' perceptions of the legal framework
Adequate rule	The standards for the certification of hospitals by the General Health Council and the chapter of hospital pharmacy of the FEUM Supplement (fifth edition 2014) were the rules mostly identified as adequate by the participants (F, S)
Rule absence	The General Health Law/Health Products Regulation. It does not recognize the pharmacist as a professional of the health team (S, A)** FEUM Supplement. Lack of legal framework for some pharmaceutical services (F)
Inadequate rule	The General Health Law/Health Supplies Regulation. The figure of the health manager (called responsible sanitario) is not suitable for the implementation of HPS (F). No difference specified between private and hospital pharmacies (S) FEUM Supplement. Lack of clarity in concepts (F). Consistency problems with the COFEPRIS pharmacy verification card (F). It is a non-binding standard (F, A). A low-profile law, consider a change in secondary legislation necessary (S, F, A, O) National Model of Hospital Pharmacy. It is voluntary (S, A). Not updated (S)
Contradictory rule	The General Health Law / Health Supplies Regulation. It only requires the health manager (called responsible sanitario) to monitor the legal sale of controlled medicines (S) FEUM Supplement. It is not aligned with the Mexican General Health Law or its Regulations (S). To achieve the changes proposed in the FEUM Supplement requires a budget (O) Regulation by COFEPRIS / Health Verification Act. Conceptual and process discrepancies with the CSG and FEUM Supplement (F) standards
Weak rule enforcement	The General Health Law / Health Supplies Regulation. Weak organizational and management capacity in hospitals, so instead of demanding compliance with the legal framework, this is only encouraged (S). Problems in supervising the assignment and fulfilling the functions of the health officer (F) FEUM Supplement. Lack of penalty for non-compliance / is not binding (F, A) Regulation by COFEPRIS / Health Verification Act. Lack of a requirement for the implementation of HPS (F, A). Staff may not be adequately trained in evaluating HPS (F)
Weak organizational capacity	At the health system level. Decentralization complicates the homogeneous implementation of new service delivery models (S). The fragmentation of the health system increases the number of service delivery models (S). The lack of hospital pharmacists at various levels of decision-making (F). Poor continuing education by universities, colleges or associations (F, A). Corruption problems in different instances (A, F, O) At the hospital level. The area to which the pharmacy service belongs influences the implementation of HPS, historically it has belonged to the area of material resources or the administrative area of the hospital (O, F). Inadequate levels of recruitment and wages for pharmacists (O, F). HPS's permanence may be subject to the decision of the hospital director (A, F). Resistance to change by members of the health team and hospital managers (F). Lack of financing for the implementation of some services (F) At the pharmacy service level. Insufficient number of hired pharmacists (O, F). The need for pharmacists with leadership and management skills (S, F). Pharmacists must have the necessary skills to develop HPS
Dysfunctional inter-organizational relationships	At the hospital level Problems of prioritization of services and activities between pharmacy staff and the hospital authority (F). Lack of hospital director support (F, A, O). Lack of support from the area manager (F) Externally Lack of adequate training of human resources in terms of HPS. Few universities train pharmacists with a focus on health sciences (O). NGOs do not make common cause on HPS (F, O). Internal struggles pharmaceutical guild produces a weak organizational capacity (S, O, A, F)

F = hospital pharmacists

S = government Stakeholders

O = representatives of non-governmental organizations

A = academics

FEUM Supplement: supplement for the establishments dedicated to the sale and supply of medicines and other health goods of the Mexican United States Pharmacopeia, fifth edition 2014. NOM-220: Official Mexican Norm Installation and operation of pharmacovigilance. CSG: General Health Council. HPS: Hospital Pharmaceutical Services. COFEPRIS: Federal Commission for Protection against Health Risks. NGO: non-governmental organization

*Categories taken from the conceptual framework proposed in Fig. 2

**This changed in December 2019, with the amendment to article 79 of the General Health Law

### Adequate rules

This category of “adequate rules” emerged during the analysis of the interviews and therefore was added to the analysis, although it was not considered in the original conceptual framework. At least one informant in each category recognized that the General Health Council (CSG) standards for hospital certification and the Pharmacopeia of the Mexican United States for its Spanish (FEUM) Supplement are the main rules related to HPS. The chapter on hospital pharmacy of the FEUM Supplement is perceived as the main rule to legally require hospital authorities to implement services that include clinical activities. However, there were still many actors who doubted the binding nature of these rules.

### Rule absence

The two groups of actors who stated that the legal framework does not have all the necessary elements for the implementation of pharmaceutical services were decision-makers and hospital pharmacists.

Some respondents identified as an obstacle that the General Health Law (LGS by its Spanish acronym) considers the practice of pharmacy as a technical activity and not as a professional service. Although this statement in the LGS was modified during the writing of this article in December 2019, it is important to note that some actors viewed the modification as only the beginning of changes needed in hospitals. As one interviewee stated:

“An article of the General Health Law is already being [developed] where they are including the pharmacist as a professional … what follows [we need] is to include the pharmacist within the organization charts of the health service … in hospitals” O13.

### Inadequate rules

The actors perceived problems in various laws related to HPS. Regarding the LGS, they pointed out that it prioritizes the regulation of medicines over the activities and services that are carried out in a pharmacy. In addition, they mentioned that the law does not differentiate between the activities and services that are organized in the retail pharmacy from those that occur in hospital pharmacies. For instance, the figure of the "health manager" (“el responsible sanitario” in Spanish) as required by law is not enough to raise the quality of care in hospitals, because legally, their functions are limited to the supervision of the sale of narcotics as a part-time activity. As one interviewee stated:

"The situation with health managers in many hospitals is: they are people who simply lend their professional license to comply and who receive a payment and nothing else. The authority must be questioned, how flexible are they in the application of the regulations [regarding the scope of the functions/responsibilities of the health manager health manager]?" F20.

In relation to the FEUM supplement (the rule that establishes the guidelines to be followed for hospital pharmacy in Mexico) related to HPS, the informants identified various problems: (1) lack of clarity in the concepts in the Hospital Pharmacy Chapter, (2) a low level of compliance without penalty, and (3) a lack of consistency between the COFEPRIS checklist for the accreditation of services by the MoH and the checklist for the certification of hospitals by the General Health Council.

“… Some pharmaceutical services are described in the FEUM supplement (2018), but it is confusing, when you read the definition… you read it again and I ask my boss and she understands something different when she reads it […]. We are pharmacists […]. Someone without preparation is not going to be able to interpret it. F17.

Regarding the National Model of Hospital Pharmacy, decision-makers and academics mentioned the importance of updating it and emphasized that its compliance is voluntary and non-binding.

### Contradictory rules

When a norm is not in line with the country context and other norms, it is considered contradictory. The pharmacists who were interviewed pointed out that the LGS allows the presence of a part-time pharmacy manager instead of being full-time, and the manager is not required to have pharmacy training:

"COFEPRIS reviews the processes, validated through a checklist … I think that the assignment of health managers, as those people who have more than 30 years as heads of pharmacy without adequate preparation, has not been so rigorous." F20.

In addition, hospital pharmacists stated that the verification process carried out by COFEPRIS shows discrepancies between the provisions of the FEUM supplement, the accreditation standards of the General Direction of Quality and Health Education of the MoH, and the quality standards for the certification of hospitals by the General Health Council. This means that pharmacists are forced to carry out different processes, depending on the situation they face, as indicated in the following statement:

"The normative framework of the hospital pharmacy has discrepancies … especially related to the regulations and the quality of care … On the one hand, we find sanitary checks, and on the other hand, the General Health Council where requests are based on a regulatory framework. All these processes are based on the legislation that govern it… But there are discrepancies in terms of concepts, there are discrepancies in how to carry them out, and one reaches the point where the discrepancies generate a lot of confusion." F20.

### Weak rule enforcement

In relation to compliance with the legal framework, the decision-makers in the Federal MoH who were interviewed mentioned that the regulatory amendments should be introduced gradually. Sudden changes could result in massive hospital closings. To incentivize compliance with regulations they suggested that requests for additional hospital funding should be conditioned upon implementation of HPS. Furthermore, they perceived that difficulties in implementing new service delivery models, such as pharmaceutical services, are related to the challenges in modernizing hospital management and the ability of health workers to achieve it, since it requires leadership. On the contrary, representatives of COFEPRIS emphasized that to achieve success in the implementation of HPS, a change in the secondary laws and their regulations is essential.

Some pharmacists are disappointed that there is no mandatory requirement for HPS implementation. In addition, they pointed out that the personnel responsible for compliance with the applicable regulations (COFEPRIS personnel) may not have the necessary training and competencies to understand the HPS implementation process.

Finally, academics agreed with NGO representatives that there is a lack of institutional accountability, so that the decision of whether to implement HPS is subject to the vision of the hospital director in charge at the time, rather than the result of an organized process designed to improve the quality of care.

### Weak organizational capacity

This category involves whether different actors involved in HPS have the necessary organizational capacity to implement, monitor and enforce the legal framework. At the national level, decision-makers pointed out that the decentralization of responsibilities of health services to the states has made it difficult for national norms and models to be implemented and monitored through a hierarchical mandate from the Federal MoH:

"The (Federal) Ministry of Health can instruct something and this instruction is not necessarily followed… this notion of some people that the Ministry of Health owns the State hospitals, that is not the reality of the 1990s; then it is not easy for the Ministry of Health to achieve a change in the Ministry of Health at the state level. It is not linear … That is why it takes so long to change health policy in our country. So, whoever thinks that health policy in our country is a matter of will is very unaware of the actual administrative and political management arrangements within the system.” S12.

Hospital pharmacists perceived problems in the organizational practice of hospitals because: (1) in many hospitals the pharmacy service depends exclusively on the administrative area, so sometimes, pharmacists trained in clinical practice cannot develop these services; (2) there is an insufficient number of hired pharmacists; (3) there is a lack of interest, time and support from the hospital authorities or state health leaders; and (4) there exists a lack of knowledge on the subject by the hospital authorities. Furthermore, hospital pharmacists pointed out the importance of hiring pharmacists with high levels of leadership and management skills.

### Dysfunctional inter-organizational relationships

Inter-organizational relationships can create problems in HPS implementation through various processes: (1) the interference of the labor union in the allocation of labor contracts and the selection of individuals to be hired for specific positions; (2) the lack of an NGO that represents the interests of hospital pharmacists; and (3) the inadequate education on hospital pharmacy provided by some universities, colleges and associations, characterized by a biased view towards pharmacovigilance and a lack of a comprehensive curriculum.

Finally, informants mentioned individual professional differences and gaps between professional pharmacy associations as one of the main obstacles for the advancement of hospital pharmacy in Mexico:

“Sometimes we are fighting over things that are not important such as if we are going to call such a definition such a thing, or if we are going to follow the model of Spain, or the model of the United States … These are sometimes silly discussions.” O3.

## Discussion

This analysis shows that the implementation of hospital pharmaceutical services in Mexico is subject, in part, to the influence of the legal framework and organizational practice; as a result, reforms in these two areas could help promote HPS implementation in the country.

Most stakeholders identified the FEUM Supplement as the document that should be used as the basis for the implementation of HPS in Mexico, although it is generally considered a voluntary rule. Because Title Twelve, Chapter I, Article 195 of the General Health Law indicates since 1984 that the FEUM and its Supplements regulate medicines and other health supplies, compliance with all the chapters of the FEUM Supplement is mandatory. COFEPRIS, as the regulatory body for health in Mexico, must supervise compliance with the FEUM supplement at the national level.

We consider that certain actions from the bottom-up, such as the support of the head of pharmacy area and the hospital director, could also work to advance HPS implementation. For example, the International Federation of Pharmacy has pointed out that keeping track of the activities and interventions carried out by the pharmacy service should be encouraged [25] because it allows one to quantitatively demonstrate the impact of the HPS. PAHO showed that keeping track of activities such as the implementation of a unit dose drug distribution system can provide evidence of cost savings [49].

It is also necessary to consider that the different elements of the Mexican legal framework related to HPS have different objectives. For example, the pharmacy health verification act (a checklist used by COFEPRIS to monitor that pharmacies have: legal and technical documentation, infrastructure, personnel and drug management in accordance with the law) should have the purpose of verifying compliance with the guidelines established in all the chapters of the FEUM supplement, while the CSG standards are intended to help improve the quality of medical and pharmaceutical care services and guarantee the safety of patients. However, to be effective, these two elements of the legal framework need to align the definitions and concepts that are core to HPS.

Organizational capacity and inter-organizational relationships must also be considered at the different stages of implementation: exploration, preparation, testing, operation and sustainability, as outlined by Moullin [50]. In this sense, the area to which the pharmacy service belongs (to a clinical area or not) can be an important factor [51]. Future research should evaluate the organizational structure of hospitals including the assignment of pharmacy staff.

The problem of differences in the perception of organizational capacity in hospitals and the lack of vigilance in complying with regulations must be systematically addressed from a top-down perspective, including the roles of: (1) a coherent legal framework; (2) the elaboration of official Mexican norms for HPS; (3) a greater and more efficient monitoring of compliance; (4) an evaluation of the quality of HPS; and (5) increased training of human resources with the necessary clinical and management skills. From a bottom-up perspective, it is important to remember that the implementation of HPS is carried out gradually according to the needs and characteristics of each hospital. To resolve conflicts between members of the hospital pharmaceutical union one possibility is to create a new and different NGO (since the first one, the Mexican Association of Hospital Pharmacists, for different reasons, only lasted 10 years and then disappeared) that can promote dialogue in Mexico among all professionals dedicated to HPS, in order to reach agreement on the core principles of HPS implementation.

The clinical and economic impacts of the implementation of HPS are an important future line of research, since these policies support the rational use of medicines and patient safety.

We consider as strengths of this study the use of semi-structured interviews to analyze the implementation of the legal framework, which allowed us to understand the perceptions of different actors. The comprehensive nature of our analysis is reflected in the broad scope of our recommendations. Given that previous studies on HPS implementation have been limited to the perceptions of hospital pharmacists, this research fills an important gap in knowledge in Mexico.

We note that when proposing changes in the legal framework and organizational practice, feasibility limitations should also be considered. This study was focused on the contextual constraints of hospitals under the authority of the MoH in Mexico. As Mathauer and Carrin suggest: "The implementation capacity of the organizations must be considered, as well as the political, technical viability … and financial sustainability" [45]*.* Therefore, future work should study the contextual factors of other health providers and consider the training capacity of institutions of higher education. The failure to include a greater variety of political actors, such as hospital directors and other health personnel, should also be considered as a limitation of this study.

Table 4 presents suggestions how to accelerate the implementation of HPS in Mexico, based on the analysis presented above, by promoting changes in the legal framework and organizational practice.

**Table 4:** Recommendations to improve the implementation of hospital pharmaceutical services through the approach of emerging bottlenecks

Rule	Current situation	Proposal for change	Possible implications
Legal framework			
General Health Law/regulation of health products	- The figure of the health manager is not suitable for the implementation of HPS - The legal framework does not differentiate between retail and hospital pharmacies	- Establish the figure of the pharmacy chief to replace the health manager - Prepare a NOM for activities and services in (at least) hospital pharmacies	- Possible resistance to change by hospital staff - Having a competent full-time pharmacy chief will increase HPS implementation
Standards for hospital certification	- They are only harmonized with the international standards of the Joint Commission; however, their implementation is not mandatory	- The critical medication management and use system can be considered as a basis for the elaboration of a NOM, from which the accreditation criteria of the Ministry of Health and the COFEPRIS pharmacy verification certificate are developed	- Having a norm based on international standards will help increase the quality of pharmacy services
The FEUM Supplement	- Absence of some pharmaceutical services - Lack of clarity in the concepts of the last edition - It is not a binding rule / a low-key law	- Establish minimum standards for all pharmaceutical services that are developed in Mexico - Review concepts based on scientific literature - Make standards binding and promote compliance	- The FEUM Supplement is the norm mostly related to the HPS, its revision, strengthening, alignment and adoption at the national level is one of the best strategies to achieve the implementation of HPS
Accreditation and certificates of medical care establishments/hospitals	- Conceptual and process discrepancies with the CSG and FEUM supplement standards - Weak enforcement of compliance - Pharmacy verification by COFEPRIS or its state offices is sometimes performed by untrained personnel for HPS evaluation	- Review, correct, evaluate and align the verification card following the appropriate standards	- A suitable regulation tool for the HPS will allow to increase the quality in the provision of these services - Make it mandatory
National Model of Hospital Pharmacy	- It is voluntary - It is not updated	- It is necessary to update and promote it	- A new effort to update it can help put the issue on the national political agenda - A NOM is recommended due to its mandatory nature
Organizational practice			
Health system level	- Compliance with the legal framework is encouraged, but not required - Problems in supervising the assignment and fulfillment of the functions of the health manager - Corruption problems in different instances - Decentralization and fragmentation of the health system complicate the standardized implementation of new service delivery models - Lack of hospital pharmacists at various levels of decision-making - Lack of financing for the implementation of some services	- Increase the mechanisms of compliance with the legal framework related to HPS. It may be necessary to increase the number of trained verifiers - Transparency of the mechanisms for assigning contracts for health managers - Promote the educational training of pharmacists with the necessary skills - Allocation of financial resources for the implementation of HPS	- Taking the impact of NOM 220 on hospital practice as an example, we consider that developing a NOM (s) for HPS will promote its implementation
Hospital level	- The implementation and permanence of HPS may be subject to the decision of the hospital director - Historically the pharmacy service has belonged to the areas of material resources or the administrative area of the hospital - Inadequate levels of recruitment and wages for pharmacists - Resistance to change by members of the health team and hospital managers - Problems of prioritization of services and activities between pharmacy staff and hospital authority	- Raise awareness among hospital directors, area managers, and health personnel about the benefits of HPS - The pharmacy service must be linked to both the medical and administrative areas - Increase the number of trained pharmacists in hospital pharmacies and assess their labor rights	- Increase in HPS implemented with the consequent decrease in costs and increase in the quality of care

It is important to note that the legal framework and organizational context for the Mexican health system in this research study (completed in March 2019) has subsequently changed:

As mentioned before, since the inception of INSABI, medicines supply has deteriorated because the federal government decided to buy medicines through direct purchases from other countries such as Argentina, without adequate verification of quality, efficacy, and safety [52]. As a result, Mexico is experiencing a significant shortage of many medicines that were previously supported by Seguro Popular, including medicines for the treatment of childhood cancer and HIV among others [53]. In addition, on August 19, 2020, COFEPRIS became part of the MoH’s Undersecretariat of Prevention and Health Promotion, by Presidential Decree [54]. The legality of this change has been questioned considering Article 17 bis of the General Health Law. It will take time to understand the impact of these significant reforms and others expected in 2021 on hospital pharmacy and the implementation of HPS. There are many important questions that will need to be investigated in future research projects.

Future studies should consider additional analysis of organizational practices, especially in private hospitals and other public health providers. In this regard, it is important to note that a change in legislation does not per se solve the gaps in implementation of health services, but contributes to the understanding of the necessary adjustments for their adoption in health systems.

## Conclusions

This study shows that the legal framework related to the implementation of hospital pharmaceutical services in Mexico exists, but it is inadequate, has been misinterpreted and requires modification. In spite of this weak legal framework, there is growing implementation of HPS in Mexico due to the organizational practice of skilled professional pharmacists, hospital directors and members of the health team. Additionally, HSP implementation has been supported by the compulsory requirement of hospitals to achieve pharmacovigilance and the certification of hospitals by the General Health Council.

To overcome the bottlenecks found in this study in the legal framework, we strongly recommend the development of a Mexican Official Norm that establishes the characteristics and minimum specifications for the pharmacy services of a hospital and implementation of HPS. To achieve this, it is necessary to take into account, among others, the critical medication management system included in the standards of the CSG and an updated National Hospital Pharmacy Model.

To resolve the bottlenecks identified in our analysis of organizational practice, we recommend that the hospital pharmacist be included as a professional in the health team, that adequate changes be made in pharmacist employment contracts, and that the number of hospital pharmacists be increased. In addition, Mexico requires a change in the perceptions of health system decision-makers (including federal and state health leaders, hospital directors, and area heads in hospitals) about the benefits of implementing hospital pharmaceutical services.

## Supplementary Information

**Additional file 1: Annex 1.** Chronology of health policy and legal framework related to hospital pharmacy in Mexico.

**Additional file 2: Annex 2.** Interview guide.

**Additional file 3: Annex 3.** CoreQ Checklist.

## Data Availability

All law, regulations and degrees reviewed for this manuscript are in the public domain. Interview transcripts will not be made available publicly since this has not been approved by the ethics review board.

## Abbreviations

COFEPRIS: Federal Commission for Protection against Health Risks
CPFEUM: Mexican United States Pharmacopeia Permanent Commission
CSG: General Health Council
FEUM: Mexican United States Pharmacopeia
INSABI: Health Institute for Wellness
LGS: General Health Law
NGO: Non-governmental organizations
NOM: Official Mexican Norm
RIS: Regulation of health products
HPS: Hospital Pharmaceutical Services
SiNaCEAM: National Certification System for Healthcare Establishments
