# Supplementary material for: The implementation of pharmaceutical services in public hospitals in Mexico: an analysis of the legal framework and organizational practice
Source: J Pharm Policy Pract. 2021 May 5;14:41. doi: 10.1186/s40545-021-00318-7 (PMC8101239; doi:10.1186/s40545-021-00318-7)
Supplement: Supplementary file 1 — Additional file 1: Annex 1. Chronology of health policy and legal framework related to hospital pharmacy in Mexico. [file 40545_2021_318_MOESM1_ESM.docx]

**Annex 1. Chronology of health policy and legal framework related to hospital pharmacy in Mexico**

| Year | Legal framework | Relationship with the hospital pharmacy |
| --- | --- | --- |
| 1983 | **Political Constitution of the Mexican United States^α^**  Art 4. The Law will define the bases and modalities for access to health services and will establish the concurrence of the Federation and the federal entities in matters of general health, in accordance with the provisions of section XVI of article 73 of this Constitution. | Primary law, on which all legal regulations are based |
| 1984 | **The General Health Law and its modifications ^β^**  Art 79. Pharmacy as a technical activity that requires legally issued diplomas  Art. 195 Of the medicines and other health supplies will be regulated by the Pharmacopoeia of the United Mexican States  Art. 198 Of the requirement of sanitary authorization the establishments dedicated to the process of medications that contain narcotic drugs and psychotropic  Art. 200 Of the operating requirements of the establishments: have a person in charge and use the latest edition of the FEUM and its supplements. Art 200 Bis. Establishments that do not require sanitary authorization must give notice of operation  Article 258 Of the requirements of pharmacies, among others, to possess and comply with the provisions of the FEUM supplements related to the sale and supply of medicines  Art. 260. Of the professional titles of the ‘health managers’ (only required if the pharmacies sell narcotic or psychotropic medications) and may be: pharmacist, biologist, pharmaceutical chemist, industrial chemical chemist, industrial chemist and physicians, or professionals whose career is pharmacy related | The General Health Law (1984), in its article 195 indicates that the Pharmacopeia of the United Mexican States regulates medications and that the Ministry of Health has the obligation to keep the FEUM updated, through a body Technical adviser named Permanent Commission of the FEUM (CPFEUM by its acronym in Spanish).  It establishes the guidelines for the management of health supplies (including medications), dividing them into controlled (narcotic and psychotropic) and over-the-counter. When a pharmacy manages the first, it must have a responsible part-time professional |
| 1992 | **First publication of the Federal Law of Metrologyand Normalization ^β^** | Following the provisions of the Federal Law of Metrology and Normalization, the official Mexican norms aim to establish the characteristics and specifications that the products and services mentioned in them must meet.  The scope and mandatory nature of the Mexican Official Standards, some of them related to the HPS, are established for the first time |
| 1997 | **First FEUM Supplement for pharmacies, drugstores, apothecaries and warehouses for the storage and distribution of medicines ^λ^** | The CPFEUM decided that private pharmacies - we call them that, because currently in Mexico the legislation does not require the presence of a full-time professional pharmacist in these establishments and only requires the presence of a part-time health manager when these establishments sell controlled medicines (narcotic and psychotropic) - they had to be professionalized and published the first supplement of the FEUM |
| 1998 | **Regulation of health products (RIS for is Spanish acronym) ^λ^**  - Chapter III. Health managers | The rule of health supplies published in 1998, describes in its third chapter the difference between pharmacies that handle controlled drugs and those that do not, indicates that the former must have a health officer. Without differentiating private from hospital |
| 2001 | **Foundation of the Federal Commission for Protection against Health Risks (COFEPRIS for its Spanish acronym) ^δ^** | Surveillance for compliance with the guidelines established by the FEUM corresponds to the COFEPRIS, which exercises the powers of regulation, control and health promotion of medicines and other compounds that constitute health risks. This agency uses a document called the health verification act for pharmacies, apothecaries and drugstores that works as a checklist to carry out health surveillance and assess compliance with applicable legislation in pharmacies |
| 2002 | First publication of the **Official Mexican Norm NOM-220-SSA1- 2002 Installation and operation of pharmacovigilance** **^Ɲ^** | It is mandatory in the national territory for health institutions and health workers |
| 2004 | **Regulations of the Federal Commission for Protection against Health Risks ^λ^**  - Article 3. Of its attributions: to exercise the regulation, control, vigilance and health promotion, that in terms of the applicable dispositions correspond to the Ministry of Health in the matter of: health establishments | Establishes that COFEPRIS is the regulatory body for pharmacies |
| 2005 | **FEUM supplement for establishments dedicated to the sale and supply of medicines and other health supplies third edition ^λ^** | Pharmacist activities in the hospital are included  - Chapter of pharmacovigilance  - Pharmaceutical Care Chapter |
| 2007 | **Health Sector Plan 2007-2012** **^λ^** | Strategy 3. Place quality on the permanent agenda of the national health system  - Line of action 3.3. Promote the rational use of medicines and the creation of clinical pharmacy units  - Line of action 3.6 Design and implement a national drug policy. implement a structuring model for hospital pharmacy in general and specialty hospitals |
| 2010 | **National Model of Hospital Pharmacy ^λ^** | The General Directorate of Planning and Development invited hospital pharmacists and academics from various higher education institutions to collaborate in the elaboration of the National Model of Hospital Pharmacy. It proposes a profound adaptation in the organization of the pharmacy in the hospitals, the personnel that compose it and the processes, both internal to the pharmacy service and to the external processes necessary to establish an operation scheme and link with the different areas of the hospital |
|  | Publication of the **FEUM Supplement for establishments dedicated to the sale and supply of medicines and other health supplies** fourth edition ^λ^ | A specific chapter was included for hospital pharmacy activities and services.  - Technovigilancy chapter  - Pharmaceutical care chapter  - Chapter of hospital pharmacy |
| 2012 | Updating of **Official Mexican Norm NOM-220-SSA1-2012 Installation and operation of pharmacovigilance ^Ɲ^** | Establishes the procedures for the installation of a pharmacovigilance committee or unit to detect and communicate to the health authority regarding adverse drug reactions |
| 2014 | **FEUM Supplement for establishments dedicated to the sale and supply of medicines and other health supplies fifth edition ^λ^** | The hospital pharmacy chapter is modified |
| 2015 | Publication of the **Norms for the certification of hospitals** by the General Health Council (CSG by its acronym in Spanish) **^λ^** | From the collaboration with the Joint Commission International®, the Homologated International Standards of the CSG were published, which have been updated in their versions from the years 2015 and 2018 and describe the handling of medications as a critical system of the hospital. Many of its guidelines coincide with those developed within the pharmaceutical services. However, quality certification by the CSG is not mandatory |
| 2016 | Updating of **Official Mexican Norm NOM-220-SSA1- 2016 Installation and operation of pharmacovigilance ^Ɲ^** | Establishes the procedures for the installation of a pharmacovigilance committee or unit to detect and report adverse drug reactions to the health authority |
| 2018 | **SiNaCEAM patient safety model. Standards to implement the model in hospitals** ^λ^ | The management and use of medications are included as a critical patient safety system |
|  | Publication of the **FEUM Supplement for establishments dedicated to the sale and supply of medicines and other health supplies, sixth edition ^λ^** | The hospital pharmacy chapter is modified and the professional responsibility chapter was amended to include a section on hospital pharmacy |
| α Primary law, β Secondary law, λ Manuals or procedures derived from secondary laws, δ Regulatory Institution  Ɲ Official Norm.  FEUM: Pharmacopeia of the United Mexican States for its Spanish acronym  SiNaCEAM: National Certification System for Healthcare Establishments | | |

**Source: Authors**
