# Supplementary material for: The implementation of pharmaceutical services in public hospitals in Mexico: an analysis of the legal framework and organizational practice
Source: J Pharm Policy Pract. 2021 May 5;14:41. doi: 10.1186/s40545-021-00318-7 (PMC8101239; doi:10.1186/s40545-021-00318-7)
Supplement: Supplementary file 2 — Additional file 2: Annex 2. Interview guide. [file 40545_2021_318_MOESM2_ESM.docx]

| **INTERVIEW GUIDE** | |  |
| --- | --- | --- |
| Interview number: | Informant password: | |
| Place and date: | | |
| Sociodemographic data  Type of informant:  Organization: | | |
| **Topics to be developed** | **Code** | **Sample question***  *The treatment of the interview was careful to include the topics. And these questions are just one example  . |
| - - First contact/approach with HPS   - Progress of HPS implementation   - Organizations / associations   - Implementation process in hospitals | Initial perception | - How was your first contact with the topic of hospital pharmacy?  - What civil society organizations do you associate with hospital pharmacy / hospital pharmaceutical services? |
| - - Definition of hospital pharmaceutical services | Definition | - Tell me, what is the hospital pharmacy for you?  - What do you know about hospital pharmaceutical services? |
| - - Opportunities in HPS implementation   - Barriers in HPS implementation | Process | - What barriers do you know for the implementation of hospital pharmaceutical services?  - What opportunities do you know for the implementation of hospital pharmaceutical services? |
| - Diversity of target group behavior | Tractability of the Problem(s) Addressed by a Statute | - How can the rules be implemented in the real context?  - Who is really involved? - Was it easy or difficult? |
| - Objectives clear and consistent - Financial resources - Hierarchical integration within and among implementing institutions. - Decision-rules of implementing agencies - Recruitment of implementing official | Ability of statute to structure implementation | - Allow / is the rule clear to achieve implementation? |
| - Support from senior executives/hospital directors/stakeholders - Commitment and leadership skill of professionals who implement a pharmaceutical service | Non-statutory variables affecting implementation | - In addition to the rules, what other factors do you consider to be involved in the implementation of hospital pharmaceutical services? |
| - Rules of HPS (knowledge and use) - Regulation - Incentives for compliance | Rules of HPS | - What rules, laws or statutes do you know about hospital pharmacy / pharmaceutical services?  - What do you think about these rules? |
