# Supplementary material for: The implementation of pharmaceutical services in public hospitals in Mexico: an analysis of the legal framework and organizational practice
Source: J Pharm Policy Pract. 2021 May 5;14:41. doi: 10.1186/s40545-021-00318-7 (PMC8101239; doi:10.1186/s40545-021-00318-7)
Supplement: Supplementary file 3 — Additional file 3: Annex 3. CoreQ Checklist. [file 40545_2021_318_MOESM3_ESM.docx]

## Annex 3 COREQ (Consolidated criteria for Reporting Qualitative research) checklist

| **Topic** | **Item No. Guide Questions/Description** | **Reported on**  **Page No.** |
| --- | --- | --- |
| **Domain 1: Research team and reﬂexivity** | | |
|  |  |  |
| *Personal characteristics* | | |
| Interviewer/facilitator | 1 Which author/s conducted the interview or focus group? | 6 |
| Credentials | 2 What were the researcher’s credentials? E.g. PhD, MD | 1 |
| Occupation | 3 What was their occupation at the time of the study? | 21 |
| Gender | 4 Was the researcher male or female? | NA |
| Experience and training | 5 What experience or training did the researcher have? | 6 |
| *Relationship with participants* | | |
| Relationship established | 6 Was a relationship established prior to study commencement? | 6 |
| Participant knowledge of | 7 What did the participants know about the researcher? e.g. personal goals, reasons for doing the research | 6 |
| the interviewer |  |  |
| Interviewer characteristics | 8 What characteristics were reported about the inter viewer/facilitator? e.g. Bias, assumptions, reasons and interests in the research topic | 6 |
| **Domain 2: Study design** | | |
| *Theoretical framework* | | |
| Methodological orientation | 9 What methodological orientation was stated to underpin the study? e.g. grounded theory, discourse analysis, ethnography, phenomenology, content analysis | 9 |
| and Theory |  |  |
| *Participant selection* | | |
| Sampling | 10 How were participants selected? e.g. purposive, convenience,  consecutive, snowball | 6 |
| Method of approach | 11 How were participants approached? e.g. face-to-face, telephone, mail,  email | 6-7 |
| Sample size | 12 How many participants were in the study? | 8 |
| Non-participation | 13 How many people refused to participate or dropped out? Reasons? | NA |
| *Setting* | | |
| Setting of data collection | 14 Where was the data collected? e.g. home, clinic, workplace | 6-7 |
| Presence of non- | 15 Was anyone else present besides the participants and researchers? | 6 |
| participants |  |  |
| Description of sample | 16 What are the important characteristics of the sample? e.g. demographic data, date | 6 y 8 |
| *Data collection* | | |
| Interview guide | 17 Were questions, prompts, guides provided by the authors? Was it pilot tested? | Annex 2 |
| Repeat interviews | 18 Were repeat inter views carried out? If yes, how many? | 6 |
| Audio/visual recording | 19 Did the research use audio or visual recording to collect the data? | 6 |
| Field notes | 20 Were ﬁeld notes made during and/or after the interview or focus group? | 6 |
| Duration | 21 What was the duration of the inter views or focus group? | 8 |
| Data saturation | 22 Was data saturation discussed? | 7 |
| Transcripts returned | 23 Were transcripts returned to participants for comment and/or | NA |
| **Domain 3: analysis and findings** | | |
| *Data analysis* | | |
| Number of data coders | 24 How many data coders coded the data? | 7 |
| Description of the coding tree | 25 Did authors provide a description of the coding tree? | NA |
| Derivation of themes | 26 Were themes identified in advance or derived from the data? | 7 |
| Software | 27 Were themes identified in advance or derived from the data? | 9 |
| Participant checking | 28 Did participants provide feedback on the findings? | NA |
| *Reporting* | | |
| Quotations presented | 29 Were participant quotations presented to illustrate the themes/findings? Was each quotation identified? e.g. participant number | 11-16 |
| Data and findings consistent | 30 Was their consistency between the data presented and the findings? | NA |
| Clarity of major themes | 31 Were major themes clearly presented in the findings? | NA |
| Clarity of minor themes | 32 Is there a description of diverse cases or discussion of minor themes? | NA |

Developed from: Tong A, Sainsbury P, Craig J. Consolidated criteria for reporting qualitative research (COREQ): a 32-item checklist for interviews and focus groups. *International Journal for Quality in Health Care*. 2007. Volume 19, Number 6: pp. 349 – 357
